# Shear wave elasticity imaging for residual endoleak and thrombus characterisation after endoleak embolisation following endovascular aneurysm repair: a canine animal study

**DOI:** 10.1186/s41747-018-0059-0

**Published:** 2018-10-10

**Authors:** Antony Bertrand-Grenier, Fatemeh Zehtabi, Sophie Lerouge, Husain Alturkistani, Claude Kauffmann, Paule Bodson-Clermont, Igor Salazkin, Hélène Héon, Guy Cloutier, Gilles Soulez

**Affiliations:** 1 Centre de recherche, Centre hospitalier de l’Université de Montréal (CRCHUM), 900 rue St Denis, Montréal, Québec H2X 0A9 Canada; 2 Laboratoire de biorhéologie et d’ultrasonographie médicale, CRCHUM, Montréal, Québec Canada; 3 Laboratoire clinique de traitement d’images, CRCHUM, Montréal, Québec Canada; 4 Département de physique, Université de Montréal, Montréal, Québec Canada; 5 Département de génie mécanique, École de technologie supérieure, Montréal, Québec Canada; 6 Département de radiologie, radio-oncologie et médecine nucléaire, Université de Montréal, Montréal, Québec Canada; 7 Département de radiologie, Centre hospitalier de l’Université de Montréal (CHUM), Montréal, Québec Canada; 8 Institut de génie biomédical, Université de Montréal, Montréal, Québec Canada

**Keywords:** Elasticity imaging techniques, Endoleak, Endovascular aneurysm repair (EVAR), Iliac aneurysm, Shear wave elastography, Ultrasonography

## Abstract

**Background:**

To evaluate residual endoleak and thrombus organisation with shear wave imaging (SWI) after endoleak embolisation through an animal study.

**Methods:**

This prospective experimental study involved eight dogs with creation of 16 iliac aneurysms and type I endoleak after endovascular aneurysm repair (EVAR). Embolisation agents were injected into the sac to seal endoleak. SWI and colour flow Doppler ultrasound (DUS) were performed at implantation, one week, and one and three months after implantation; for three dogs, SWI and DUS were also performed six months after implantation. Digital subtraction angiography and contrast-enhanced computed tomography were performed at sacrifice. Macroscopic and histopathological analyses were processed to identify regions of interest (ROIs) for endoleak, fresh thrombus, organised thrombus and embolisation agent, where SWI elasticity moduli were compared.

**Results:**

At sacrifice, nine aneurysms had residual endoleak, while seven were sealed. Ten had a fresh and 15 had an organised thrombus. SWI was able to detect all endoleaks, including two cases undetected with DUS. Elasticity moduli of 0.2 kPa ± 0.1 kPa (mean ± SD), 9.5 kPa ± 3.3 kPa, 48.1 kPa ± 21.3 kPa and 44.9 kPa ± 23.7 kPa were found in the ROIs positioned in endoleaks, fresh thrombi, organised thrombi and embolisation agent, respectively. Elasticity values of endoleak and fresh thrombus were lower than those of organised thrombi and embolisation agent (*p* < 0.001). Stiffness of fresh thrombus at one week (8.7 kPa ± 3.6 kPa) increased at three months (30.2 kPa ± 13.8 kPa), indicating thrombus maturation (*p* < 0.001).

**Conclusions:**

In a dog model of iliac EVAR, SWI was able to identify endoleak, thrombus maturation and embolising agents after endoleak embolisation.

## Key points

- SWI is able to identify residual endoleak after embolisation
- SWI can characterise the elasticity of embolisation agents used for endoleak
- SWI can characterise thrombus organisation over time after endoleak embolisation, distinguishing between fresh and organised thrombi

## Background

Endoleak is the main complication of endovascular aneurysm repair (EVAR) requiring lifelong follow-up with computed tomography (CT) or Doppler ultrasound (DUS) [1]. Follow-up with CT leads to the potential nephrotoxicity of iodined contrast and a not negligible cumulative exposure to ionising radiation, with an attributable cancer risk estimated to be 0.65% for a 55-year-old patient [2, 3]. In this setting, DUS is recommended after one-year CT follow-up, if there is no evidence of endoleak [4]. However, DUS sensitivity is limited in particular for the detection of type II endoleak [5]. Better sensitivity can be obtained using contrast-enhanced ultrasound [5], but its clinical use is still limited, in particular in North America because it requires a specific training, an intravenous access, additional examination time and cost related to contrast agent. There are few data in the literature on the relationship between thrombus organisation, endoleak and aneurysm shrinkage. Magnetic resonance imaging (MRI) has shown that patients with an endoleak or endotension have areas of non-organised thrombi [6].

Embolisation with thrombin, colis, or liquid agent (ethylene vinyl alcohol copolymer, cyanoacrylates) has been proposed to prevent or treat endoleaks [7]. However, despite embolisation, endoleak recurrences are frequently observed [7, 8] and the role of endothelial lining in endoleak formation and recurrence was established [9]. To improve the results of endoleak embolisation, an injectable chitosan hydrogel combined with a sclerosing agent (sodium tetradecyl sulfate [STS]) was developed to promote endothelial ablation and fibrous healing of the sac [8, 9].

The potential of shear wave imaging (SWI) to detect endoleak and characterise thrombus organisation after EVAR was previously reported in a canine model [10]. Our goal was to evaluate, always in a canine model, the potential of SWI to characterise aneurysm healing and thrombus maturation over time after endoleak embolisation using embolising gels with or without sclerosing agents.

## Methods

Animal procedures were approved by our institution’s Animal Care Committee in accordance with Canadian Council on Animal Care guidelines. The method for the creation of occlusive and sclerosing gels and their safety/efficacy to treat endoleak was previously reported including six animals of the current study [8]. The current publication focus on the potential of SWI for characterising aneurysm healing after endoleak embolisation. The first draft of this article is available on a university repository website (https://papyrus.bib.umontreal.ca/xmlui/bitstream/handle/1866/16033/Bertrand-Grenier_Antony_2015_these.pdf?isAllowed=y&sequence=6).

### Creation of bilateral iliac aneurysms

Eight mongrel dogs (25–50 kg of bodyweight) underwent surgical construction of bilateral aneurysms in the common iliac arteries (16 aneurysms in total) using a venous patch taken from the external jugular vein. A collateral vessel (branch division of the sacral artery) was re-implanted in the aneurysm as previously reported [11]. Procedures were performed by a vascular surgeon with 20 years of experience in experimental surgery (I.S.).

### EVAR with type I endoleak creation

After eight weeks of recovery, EVAR was performed by an interventional radiologist with a 22 years of experience (G.S.) using a 59-mm-long balloon-expandable stent-graft (iCAST, Atrium, Hudson, NH, USA) deployed to a diameter of 7 mm or 8 mm. A type I endoleak was then created in all aneurysms by inflating a 3-mm diameter balloon catheter alongside the proximal landing zone of the stent-graft after deployment, creating a misfit between the stent-graft and the vessel wall [12].

### Endoleak embolisation

The injectable chitosan hydrogel (Chi) was prepared by mixing an acidic solution of chitosan containing a radiopaque agent with beta-glycerophosphate [13], with only occlusive property. A second occlusive and sclerosing gel formulation was obtained combining Chi with sodium tetradecyl sulfate (Chi-STS) [13]. Both embolisation agents were slowly injected in the same animal, with random side attribution, in a blind fashion for the type of gel (Chi or Chi-STS), under fluoroscopy through a 4-French catheter positioned alongside the stent-graft (Glidecath, Terumo, Tokyo, Japan). The operator was asked to occlude the entire aneurysm sac while avoiding gel migration in the stent-graft lumen.

### Angiography

Percutaneous transfemoral angiography (Koordinat 3D II, Siemens Healthineers, Erlangen, Germany) was performed after stent-graft implantation, endoleak embolisation and before sacrifice at three months (*n* = 5 dogs) or six months (*n* = 3 dogs). Type I endoleak was defined as residual opacification of the aneurysm through an antegrade flow coming from the proximal neck and type II endoleak as a retrograde flow coming from the collateral vessel [14]. CT was the reference standard for endoleak detection while angiography was only used to classify endoleaks.

### Computed tomography

CT was performed before sacrifice and reviewed by the same interventional radiologist. Arterial and venous phases were acquired, with a retrospective electrocardiographic gating reconstructed at 70% of the RR interval (60 mL at 4 mL/s, Omnipaque 300 mg I/mL, General Electric Healthcare Canada, Mississauga, ON, Canada) with acquisition parameters set at 120 kVp and 724 mAs (Somatom Sensation 64, Siemens Healthineers, Forcheim, Germany). Endoleaks were characterised as areas of contrast enhancement in the aneurysm sac visible in the arterial or venous phase.

### Ultrasound

A research technician with 20 years of experience performed independently DUS and SWI examinations at one week, one month, three months and six months (only three dogs at the last time point). Post-processing of SWI, segmentation and registration of region of interests (ROIs) on imaging acquisition and pathology examination were performed by a PhD student in medical physics (A.B.G.).

A 256-element linear probe (SuperLinear™ SL15–4, 7.5 MHz) was used for all B-mode, DUS and SWI acquisitions (Aixplorer, Aix-en-Provence, France). Three axial acquisition planes were taken on the aneurysm (proximal, middle and distal). The diameters and areas of the three axial planes were measured and averaged. Aneurysm growth or shrinkage was estimated as the variation in percentage of the mean aneurysm cross-sectional surface area between baseline and follow-up.

Standard parameters for DUS examinations to detect endoleak were set to a scale of 10 cm/s, smoothing to 0, wall filter to low, and high-definition frame rate to middle. The steer angle was first set to 0° then to 60° right anterior oblique and left anterior oblique.

Dynamic elastography parameters were selected as a smoothing of 5, opacity of 50% and low acoustic power. For elasticity measurement, the colour code scale displayed on SWI images was converted to grayscale after calibration. SWI was used to observe the evolution of the mechanical property of the thrombus and embolisation agent over time. Endoleak on SWI was defined as the absence of elasticity values within the aneurysm sac outside the stent-graft with the presence of signal on the posterior wall [10]. Areas without signal on the posterior wall were deemed as non-diagnostic.

### Pathology

Each dog was sacrificed with a barbiturate overdose (108 mg/kg, euthanyl forte, Bimeda-MTC Animal Health Inc., Cambridge, ON, Canada). Aneurysms were collected and fixed in buffered formalin. Consecutive 3–5 mm axial macroscopic sections were prepared with a cutting-grinding system to keep the implant/tissue interface intact (EXAKT Advanced Technologies GmbH, Norderstedt, Germany). In some samples, the SG was removed and tissues were sent to histology. Macroscopic cuts and histology analyses were processed independently by a PhD student (F.Z.) supervised by a biomedical engineer with 15 years of experience in endovascular biomaterials (S.L.) to identify and segment the different ROIs. Endoleaks were identified as defect zones, organised thrombus as a dense tissue with a yellowish colouration, fresh thrombus as areas with dark bluish loose tissue and embolisation agents as yellow/pink areas without appearance of tissue organisation. Macroscopic cuts were used as the reference standard for ROI segmentation and to characterise thrombus organisation and agent degradation.

Histopathologic examination was performed to confirm the content of the different ROIs assessed on macroscopic results.

### ROI segmentation and co-registration

At sacrifice, axial images acquired on B-mode ultrasound, DUS, SWI, CT and histopathology were co-registered based on the level of acquisition, aneurysm area and stent-graft localisation in the sac. TeraRecon (AquariusNET iNtuition, version 4.4.7, TeraRecon Headquarters, Foster City, CA, USA) and Image J (Rasband, W.S., version 1.47b, National Institutes of Health, Bethesda, MD, USA) were used to segment ROIs of the aneurysms.

Presence of contrast enhancement on CT scans combined with the presence of a defect in the aneurysm sac on the macroscopic examination was used as the reference standard for endoleak diagnosis [10].

For ultrasound examinations acquired before sacrifice at one week, one month and three months (for the three dogs with six-month follow-up), on each acquisition plane, the aneurysm was divided into three different ROIs: endoleak; total thrombus; and embolisation agents. ROI segmentation and positioning were based on the fusion of B-mode, DUS and elasticity images which were acquired at the same level.

Endoleak ROIs were traced based either on colour DUS or SWI examinations. Total thrombus was defined as ROIs without endoleak or embolising agent in the aneurysm sac. The ROI segmentation of embolising gel was based on the echogenicity of the gel on B-mode ultrasound which was hyperechoic compared to native thrombus. In the case of discordance or uncertainty for embolising gel contouring, the operator relied on the gel localisation on pathologic examinations.

To determine stiffness values corresponding to different levels of thrombus organisation, the thrombus was sub-classified in fresh or organised thrombus on the pre-sacrifice examination. To evaluate fresh thrombus maturation over time, ROIs with elasticity values compatible with the presence of a fresh thrombus (in the range of 3–19 kPa as previously reported [10]) on the one-week SWI examination were segmented and registered on the three-month SWI examination.

The different ROIs elasticity measurements were averaged for the three axial acquisition planes. The percentage of growth or shrinkage of aneurysms between the one-week time point and sacrifice was correlated with the averaged surface area of endoleak and/or fresh thrombus found at sacrifice on pathology (Pearson coefficient). Thresholds for significance of *p* values were adjusted with Bonferroni correction by testing each individual hypothesis at a significance level of α/*m*, where α is the desired overall alpha level and *m* is the number of hypotheses. The overall significant level was α = 5% and the significant level of comparison for multiple testing was also 5%. Statistical analyses were performed using R software (version 3.2.1, R Foundation for Statistical Computing, Vienna, Austria).

## Results

No complications occurred during procedures and imaging acquisitions. At sacrifice, among the 16 aneurysms, nine had type I endoleaks (six in the Chi group and three in the Chi-STS group) and seven were completely sealed (two and five, respectively). There was a trend toward less residual endoleak with Chi-STS, however not reaching the significance (*p* = 0.072). Fresh thrombus was observed in ten aneurysms (six aneurysms with endoleak and four sealed), while 15 had organised thrombus. There was one spontaneous endoleak seal during follow-up. In four cases of residual endoleaks (three in the Chi group, one in the Chi-STS group), no endoleak had been initially detected with angiography suggesting possible cases of recurrence.

### DUS, B-mode ultrasound, and SWI

**Fig. 1:**
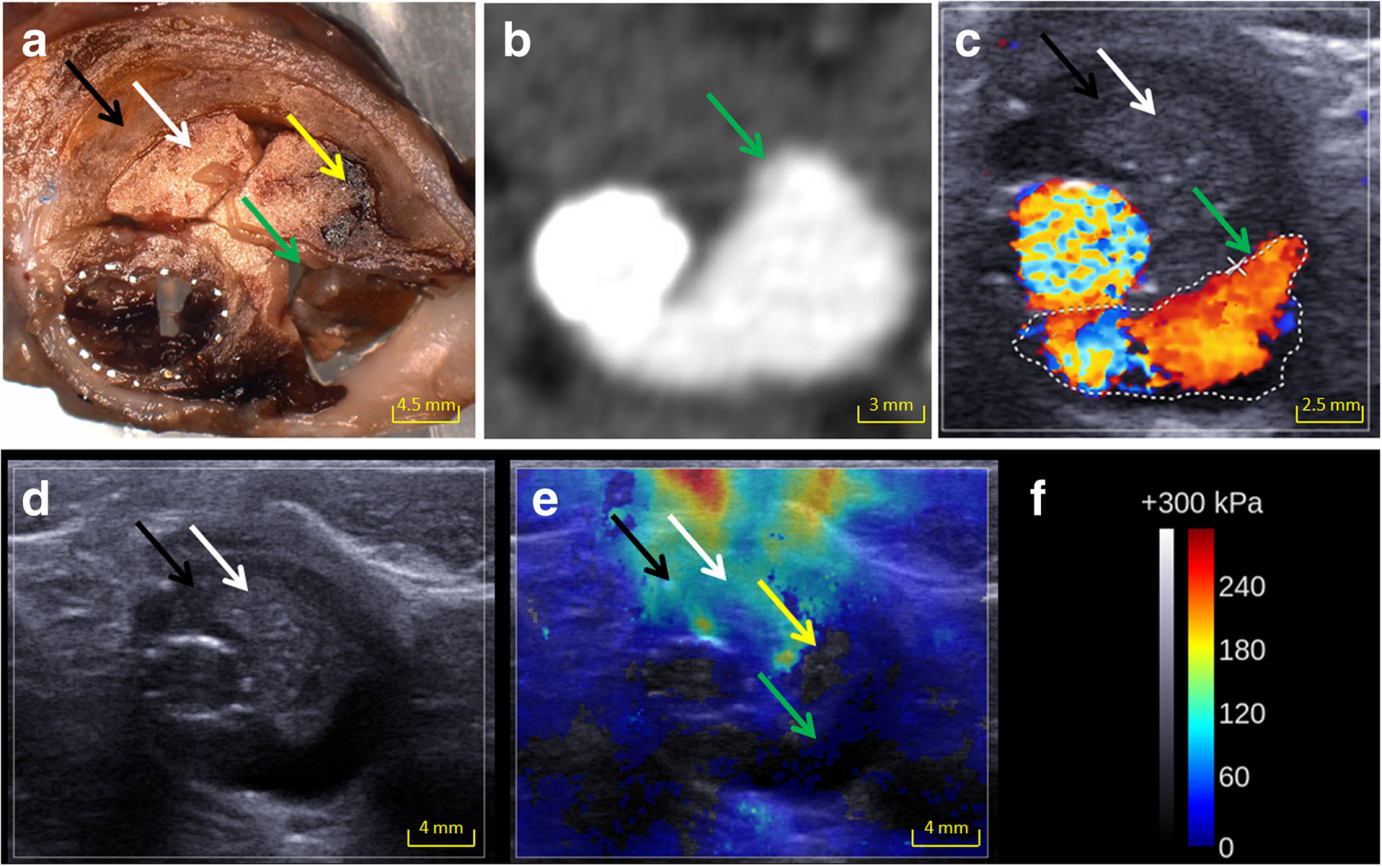
Images from an aneurysm with endoleak (*green arrow*), chitosan (*white arrow*), less-organised chitosan (*yellow arrow*) and organised thrombus (*black arrow*). **a** Macroscopic cut showing the different regions of interest. **b** CT scan showing the endoleak. **c** Endoleak on DUS. **d** Areas filled with chitosan were slightly hyperechoic and areas with endoleak were anechoic on B-mode ultrasound. **e** Endoleak and less organised chitosan were visualised on SW. **f** Colour scale and Q-Box values for SWI

**Fig. 2:**
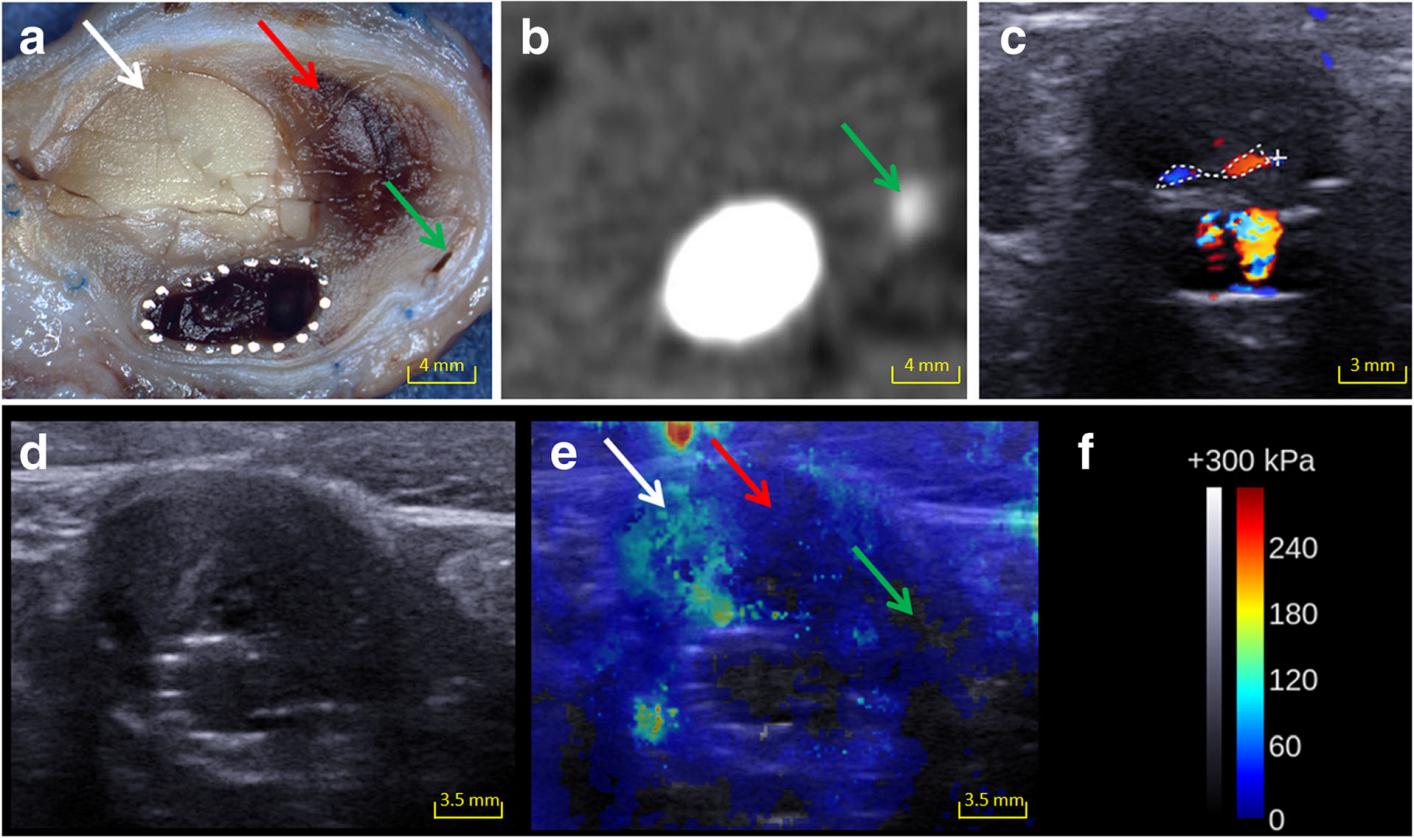
Images from an aneurysm with endoleak (*green arrow*), chitosan (*white arrow*) and massive fresh thrombus (*red arrow*). **a** Macroscopic cut showing the different regions of interest. **b** The small endoleak is depicted on CT scan. **c** DUS showing the endoleak. **d** On B-mode ultrasound, the chitosan is seen as a hyperechoic area but endoleak area is not clearly depicted. **e** The regions of interest corresponding to chitosan, soft thrombus and endoleak are depicted on SWI. **f** Colour scale and Q-Box values for SWI

All endoleaks were clearly seen on SWI as an area of minimal or no elasticity value (< 1.5 kPa) (Figs. 1 and 2), whereas DUS failed to detect an endoleak in two out of nine cases. It was not possible to differentiate fresh and organised thrombi on B-mode ultrasound. Areas filled with embolisation agents were slightly hyperechoic and were detected on B-mode ultrasound (Figs. 1 and 2).

### Elasticity measurements at sacrifice

Examples of co-registered macroscopic cut, CT scan, DUS and SWI measurements are provided in Figs. 1 and 2. The elasticity moduli of all selected ROIs are summarised in Table 1, while Table 2 presents statistical analyses.

**Table 1:** Mechanical properties of the regions of interest according to the time points

Region of interest	1 week	1 month	3 months	6 months
Endoleak	0.2 ± 0.3 (*n* = 6)	0.0 ± 0.1 (*n* = 7)	0.2 ± 0.2 (*n* = 9)	0.1 ± 0.2 (*n* = 3)
Fresh thrombus	Not performed	Not performed	9.2 ± 2.7 (*n* = 7)	10.2 ± 5.3 (*n* = 3)
Organised thrombus	Not performed	Not performed	55.4 ± 21.2 (*n* = 15)	33.6 ± 13.7 (*n* = 5)
Total thrombus	39.6 ± 21.3 (*n* = 15)	52.4 ± 21.3 (*n* = 15)	45.6 ± 22.2 (*n* = 15)	28.8 ± 12.9 (*n* = 5)
Chitosan	27.7 ± 6.8 (*n* = 8)	46.1 ± 9.6 (*n* = 8)	45.9 ± 20.7 (*n* = 8)	33.5 ± 18.2 (*n* = 3)
Chitosan-STS	36.2 ± 20.9 (*n* = 8)	52.9 ± 38.2 (*n* = 8)	49.1 ± 30.9 (*n* = 8)	30.5 ± 7.7 (*n* = 3)

Data are kPa expressed as mean ± standard deviation. *n* indicates the number of aneurysms

**Table 2:** Tukey comparisons of the mechanical properties of the regions of interest at sacrifice at three or six months

Comparison of elasticity values	kPa (mean ± standard deviation)	*p* value
Endoleak vs fresh thrombus	0.2 ± 0.1 vs 9.5 ± 3.3	< 0.001
Endoleak vs organised thrombus	0.2 ± 0.1 vs 48.1 ± 21.3	< 0.001
Endoleak vs agents	0.2 ± 0.1 vs 44.9 ± 23.7	< 0.001
Fresh thrombus vs organised thrombus	9.5 ± 3.3 vs 48.1 ± 21.3	< 0.001
Fresh thrombus vs agents	9.5 ± 3.3 vs 44.9 ± 23.7	< 0.001
Organised thrombus vs agents	48.1 ± 21.3 vs 44.9 ± 23.7	1.000

Note: all elasticity values were compared on animal sacrificed at three months (*n* = 5) or six months (*n* = 3). Values were log-transformed to better meet normality assumptions. Comparisons were significant overall in the ANOVA results (*p* < 0.001), there was no influence of the time at sacrifice (three or six months) on elasticity values (*p* = 0.275) and type of gel (Chi or Chi-STS; *p* = 0.713)

The elasticity values of endoleaks and fresh thrombi were significantly lower than other ROIs, including organised thrombi (*p* <  0.001), but elasticity of ROIs containing organised thrombi and embolising agent were similar. Results remain consistent when adjusting for type of agent (Chi or Chi-STS) and time of sacrifice (three months or six months).

### Evolution of thrombus and embolising agents’ elasticity over time

Elasticity values of the total thrombus ROIs showed a trend for increase between one week and one month but there was no significant effect of time (*p* = 0.111) or type of agent on thrombus elasticity (*p* = 0.618). Embolisation agent elasticity also showed a trend for increase over time. Similarly, there was no significant effect of time (*p* = 0.118) or type of agent (*p* = 0.769) on embolisation agent stiffness.

### Evolution of fresh thrombus elasticity over time

The time analysis showed that areas tagged as fresh thrombus at one week (ROIs showing a range of 3–19 kPa) were stiffer at three months (30.2 kPa ± 13.8 kPa) when compared to baseline at one week (8.7 kPa ± 3.6 kPa) due to thrombus organisation (*p* < 0.001). This maturation over time was not significantly different for both agents (*p* = 0.358) (Table 1). In 12 aneurysms, fresh thrombus ROIs evolved to organised thrombus at three months, while in four aneurysms (two embolised with Chi and two with Chi-STS), the elasticity in the same region was still in the range of fresh thrombus values. None of these four aneurysms had a residual endoleak.

### Aneurysm size as a function of endoleak and fresh thrombus

**Fig. 3:**
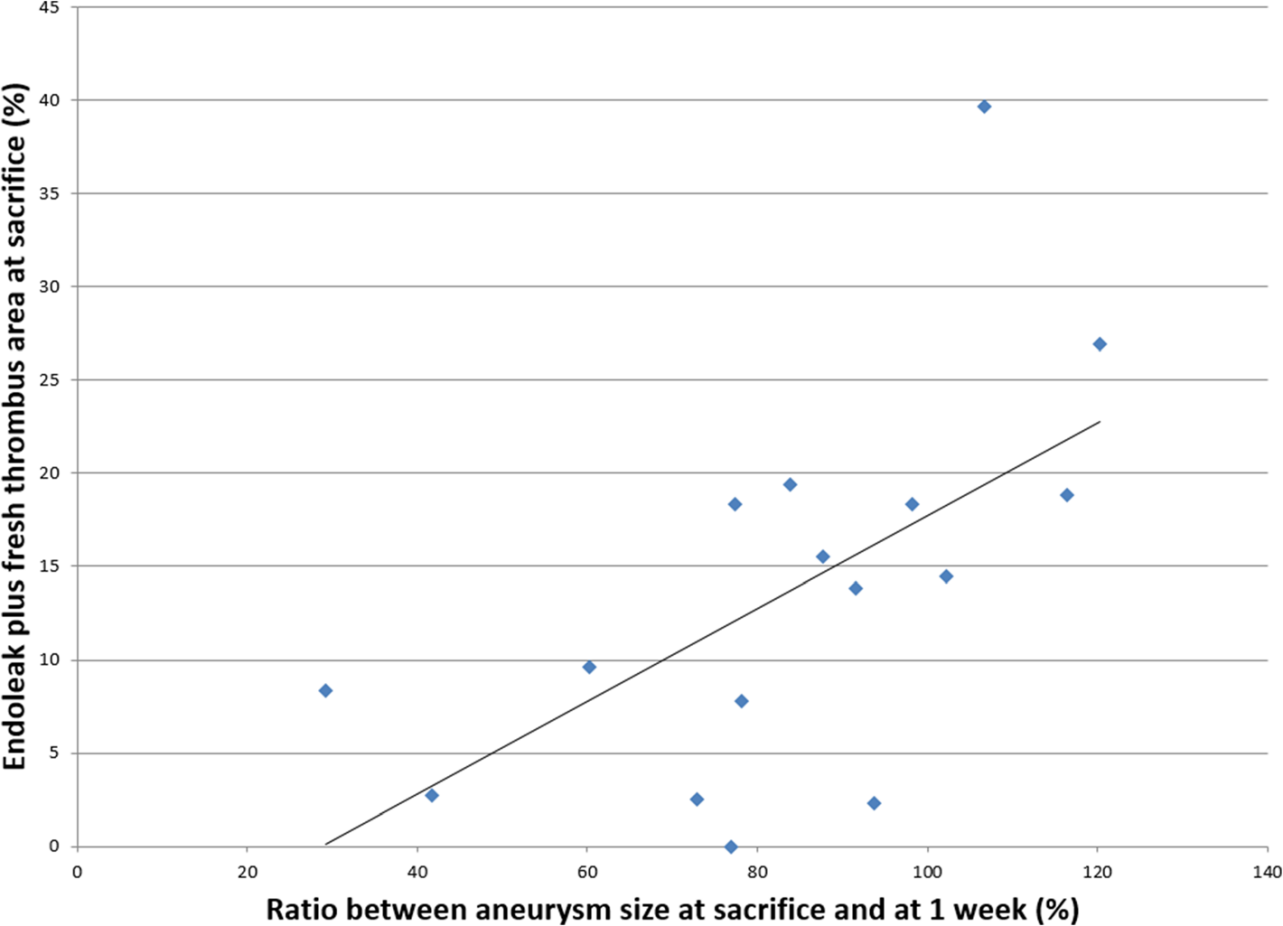
Evolution of aneurysm size as a function of endoleak and fresh thrombus areas at sacrifice. Pearson coefficient *r* = 0.646, *p* = 0.007

The correlation of aneurysm growth and surface area of ROIs containing endoleaks or fresh thrombi on pathology is shown in Fig. 3. There was clearly less aneurysm shrinkage when the surface area of endoleaks or fresh thrombi increased (*r* = 0.646; *p* = 0.007).

## Discussion

This study confirms that SWI is able to characterise thrombus organisation and embolisation agents over time following EVAR. This imaging technique consistently provided the elastic modulus within the different ROIs. As previously reported in a primary endoleak model, SWI can detect endoleak with a high sensitivity [10]. In the current study, its potential to detect residual endoleak after embolisation is confirmed. In addition, elasticity measurement could be useful in providing an assessment of the fibrous organisation of the aneurysm sac after embolisation. Thus, this technique has the potential to monitor thrombus maturation over time.

In the current clinical practice, patient follow-up after endoleak embolisation relies on endoleak detection and measurement of aneurysm maximal diameter. Elasticity measurement could be an additional tool, particularly for patients having embolisation with highly radio-opaque agent (coil, ethylene vinyl alcohol copolymer) impairing endoleak detection on CT scans.

While liquids do not support shear wave propagation, endoleaks display no elasticity value [15]. This is confirmed by the very small (probably artefactual) values we observed in endoleak areas. With respect to fresh thrombus, since it is made mainly of fibrin clot, it appeared softer on SWI examinations. A lower elastic modulus corresponds to fresh and soft thrombi, whereas a higher modulus corresponds to more mature and rigid thrombi. Therefore, stiffness measurements can be used to classify the organisation of a thrombus [16–18] and SWI could provide new information that cannot be obtained on CT scans or standard ultrasound (B-mode or DUS).

Regarding, the characterisation of embolising agents, we should consider that Chi and Chi-STS gels had elasticity values in the same range as organised thrombus. Even with liquid agents such as Onyx or glue, up to now, the results of endoleak embolisation have been disappointing [7]. In the current study, the combination of a sclerosing agent with chitosan (Chi-STS) showed a trend for decreasing endoleak persistence. However, probably because of the small sample size, this effect did not reach the statistical significance. The better efficacy of Chi-STS over only Chi gels was previously reported [8]. SWI could be used to monitor the stiffness and biodegradation of new generation embolisation agents.

The stiffness of total thrombus and both embolising agents tended to increase with time; however, this did not reach significance. This was probably related due to high measurement variability in the ROIs between animals and the small sample size (Table 1). We observed an overall increase in fresh thrombus stiffness over time in 12 out of 16 aneurysms. Aneurysm sacs without endoleak showing a progressive increase in thrombus stiffness are probably at lower risk of developing a delayed endoleak or endotension. Conversely, low thrombus stiffness with or without endoleak is probably indicative of a higher risk for aneurysm growth or delayed endoleak. The detection of this unorganised, less rigid thrombus could also be linked with endotension or very slow-flow endoleak not detected on CT scans or DUS [6, 19].

Chi-STS which combine occlusive and sclerosing effects was developed to promote endothelial ablation and fibrous healing of the aneurysm [13]. With respect to the effect of the type of agent on thrombus stiffness, we did not find significant differences with SWI. Thus, we cannot conclude if the presence of agent influences the fibrous organisation of the thrombus or if the SWI technique is sensitive enough to detect subtle differences in thrombus organisation. In a previous publication reporting the efficacy of both gels, we did not notice significant differences on histology scores between aneurysm embolised with Chi or Chi-STS [8].

We observed a positive correlation between the size of ROIs containing an endoleak or a fresh thrombus and aneurysm growth. After EVAR, fresh thrombi characterised as hyperintense areas on T1-weighted or T2-weighted MRI acquisition were observed more frequently in non-shrinking aneurysms [6, 19]. SWI could be an interesting alternative given the limited availability of MRI and the presence of metallic artifacts when stent grafts made of stainless steel are used [20].

The potential clinical application of our results should consider the current practice of patient follow-up after EVAR. Ultrasound is recommended in this clinical setting [21]. After endoleak embolisation, a close CT surveillance with contrast injection is usually performed [22]. However, endoleak detection can be challenging on CT due to the high density of embolic materials. Contrast-enhanced ultrasound is very sensitive to detect endoleak after EVAR and can be a good alternative [23]. Other ultrasound elasticity techniques such as pulse wave imaging or strain analysis were previously reported in preclinical and clinical studies to assess the wall stiffness of abdominal aortic aneurysms [24, 25]. Only one study reported its feasibility in the context of EVAR follow-up [26]. Ultrasound elasticity imaging by SWI or acoustic radiation force imaging is now available on ultrasound units and can be easily integrated in the imaging surveillance of patients following endoleak embolisation. It has the ability not only to detect endoleak but also to bring another biomarker of aneurysm healing by assessing thrombus and embolisation agent elasticity.

Our study has limitations. First, to compare both embolisation agents in the same animal, the aneurysms were created in the iliac arteries instead of in the abdominal aorta. As a consequence, the diameters of aneurysms (about 2–3 cm) were smaller than those observed in humans in the aorta. Second, this experimental setting did not reproduce the context of atherosclerotic disease. Third, the complex process of intermodal image co-registration and correlation with histology could have been source of small sampling errors. Fourth, we did not perform *ex-vivo* ultrasound elasticity imaging on the samples because the boundary conditions would have been radically different. Fifth, the linear ultrasound probe used had a maximum depth penetration of 5–7 cm while in a clinical context on humans, a phased curved array probe with a lower frequency to image the far field would be required. Sixth, we observed a large variability of elasticity measurements among subjects and did not evaluate intra-/inter-observer variability; this will be addressed in an undergoing first study on humans [27]. Seventh, the diagnosis of endoleak using SWI was based on the detection of a liquid area without elasticity. Thus, it could be challenging to detect an endoleak in a very fresh thrombus observed just after aneurysm sac exclusion. Indeed, in the peri-operative period, contrast-enhanced ultrasound could be better suited than SWI to detect endoleak.

In conclusion, the present work suggests that SWI could complement current imaging modalities by adding the possibility of grading elasticity to characterise thrombus organisation, embolisation agents and healing of aneurysms after endoleak embolisation. This information is not provided by CT scans. This real-time ultrasound imaging modality can complement conventional DUS and can also be combined with contrast-enhanced ultrasound, which has an excellent sensitivity to detect endoleak.

## Abbreviations

Chi: Chitosan hydrogel
Chi-STS: Chitosan hydrogel plus sodium tetradecyl sulfate
CT: Computed tomography
DUS: Colour flow Doppler ultrasound
EVAR: Endovascular aneurysm repair
MRI: Magnetic resonance imaging
ROI: Region of interest
SWI: Shear wave imaging
